# COVID-19 FAQs in Pediatric Cardiac Surgery: 2022 Perspective and Updates

**DOI:** 10.1177/21501351221085966

**Published:** 2022-05

**Authors:** Emily R. Levy, Joseph A. Dearani, Jennifer Blumenthal, Jonathan N. Johnson, David M. Overman, Elizabeth H. Stephens, Kathleen Chiotos

**Affiliations:** 1 Divisions of Pediatric Infectious Diseases and Pediatric Critical Care Medicine, Department of Pediatric and Adolescent Medicine, 6915 Mayo Clinic, Rochester, MN, USA; 2 Department of Cardiovascular Surgery, 6915 Mayo Clinic, Rochester, MN, USA; 3 Mayo Clinic-Children’s Minnesota Cardiovascular Collaborative, Rochester and Minneapolis, MN, USA; 4 Division of Critical Care Medicine, Department of Anesthesiology, Critical Care and Pain Medicine, 1862 Boston Children’s Hospital, Boston, MA, USA; 5 Division of Infectious Diseases, Department of Medicine, 1862 Boston Children’s Hospital, Boston, MA, USA; 6 Division of Pediatric Cardiology, Department of Pediatric and Adolescent Medicine, 6915 Mayo Clinic, Rochester, MN, USA; 7 Division of Cardiovascular Surgery, Children’s Minnesota, Minneapolis, MN, USA; 8 Divisions of Infectious Diseases and Critical Care Medicine, 6567 Children’s Hospital of Philadelphia, PA, USA

**Keywords:** COVID-19, vaccination, congenital heart disease, pandemic, congenital heart surgery

## Introduction

The COVID-19 pandemic continues to change the world. The healthcare industry must adapt and respond as we understand more about the detection, transmissibility, severity of illness, vaccination, quarantine and isolation duration, effects on children, and treatment options for SARS-CoV-2, including variants of concern. While pediatric heart surgery (and most surgical subspecialties for that matter) is running at or near normal capacity without significant restrictions, important issues in pediatrics continue to evolve. Since our original publications (below), several variants of concern have appeared, vaccinations have been authorized for children ≥ 5 years old, advances in diagnosis and treatment have occurred, and Multisystem Inflammatory Syndrome in Children (MIS-C) has emerged as an important entity. In light of these developments, an updated document is needed to ensure clarity of understanding and to facilitate optimal clinical decision-making.^1,2^

1. Are COVID-19 vaccinations recommended for pediatric congenital heart surgery patients?

Yes, all eligible patients should be vaccinated against COVID-19 per the Centers for Disease Control and Prevention (CDC) and Advisory Committee on Immunization Practices (ACIP) schedule. Clinicians should be aware that the number of recommended doses may differ between immunocompromised and immunocompetent patients. For immunocompromised children, including cardiac transplant patients, there are additional doses recommending in the primary series for most ages. In addition, guidance surrounding administration of booster vaccines is evolving and clinicians should check the ACIP website for the most updated recommendations (ACIP COVID-19 Vaccine Recommendations | CDC). As of January 2022, only the Pfizer/BioNTech mRNA vaccine is available for children in the United State, but there are ongoing clinical trials evaluating other pediatric vaccine candidates. Pediatric vaccine trials and observational studies have consistently demonstrated the COVID-19 vaccine is both safe and effective, with >90% effectiveness against severe disease.^3–5^ Children with underlying comorbidities, such as congenital heart disease, are at higher risk for severe disease, highlighting the importance of vaccination in this vulnerable group.

https://www.cdc.gov/vaccines/covid-19/clinical-considerations/covid-19-vaccines-us.html

2. Are there any specific considerations related to vaccine timing in cardiac surgery patients?

As above, all eligible congenital heart disease patients should receive the COVID-19 vaccination. Ideally, the vaccine series should be completed at least two weeks prior to major surgery to reduce the chance of a child acquiring severe COVID-19 in the peri-operative period, but surgery should not be delayed in order to complete the vaccine series. In particular, patients who are candidates for heart or lung transplants should receive the COVID-19 vaccination series at least 2 weeks prior to transplant whenever possible to optimize protection both pre- and post-transplant (see question 7). This highlights the important role of cardiac surgeons in advocating for vaccination during pre-transplant evaluations.

Unvaccinated patients hospitalized for cardiac surgery or other illness should be strongly considered for vaccination prior to hospital discharge. Neither of these scenarios are themselves contraindications to vaccination; however, it is prudent to wait until after recovery from major surgery and critical illness to optimize immunogenicity from the vaccine.^6^ Cardiopulmonary bypass and transfusions may dilute or suppress the patient's intrinsic immunologic vaccine response. For these reasons, we often suggest vaccination immediately prior to discharge.

https://ishlt.org/ishlt/media/documents/ISHLT-AST-ASTS_Joint-Statement_COVID19-Vaccination_30-December.pdf

3. What are the contraindications to pediatric COVID-19 vaccination?

There are very few absolute contraindications to pediatric COVID-19 vaccination. Contraindications include a severe allergic reaction (eg, anaphylaxis) to a prior dose of COVID-19 vaccine or vaccine component and/or history of COVID-19 vaccine-related myopericarditis (discussed below in question 4).

Nonsevere reactions or allergies to the COVID-19 vaccine components, other vaccines, or injectable therapies, are precautions for COVID-19 vaccines; a detailed discussion of these is available on the CDC website (https://www.cdc.gov/vaccines/covid-19/clinical-considerations/covid-19-vaccines-us.html#Contraindications). Allergic reactions (including anaphylaxis) that are unrelated to the vaccine (eg other medications, animals, venom, food, environmental allergies) are no contraindications or precautions. Some patients may experience a significant local reaction including erythema and induration after vaccination; this is not a contraindication to subsequent COVID-19 vaccines.^7^

Several myths without any scientific evidence have circulated about the COVID-19 vaccine. The authors recommend the ACIP website below for up-to-date factual content. For instance, there are no contraindications to vaccination related to fertility or future or current pregnancy. On the contrary, it has been demonstrated that pregnant women who are not vaccinated are at higher risk of severe COVID-19 which may lead to fetal demise or preterm delivery. In addition to trial safety data, the United States has an extensive system to detect rare vaccine side effects not identified in trials.

ACIP COVID-19 Vaccine Recommendations | CDC

https://www.cdc.gov/vaccines/covid-19/clinical-considerations/covid-19-vaccines-us.html

COVID-19 Vaccination Considerations for Obstetric–Gynecologic Care | ACOG

4. Does the COVID-19 vaccine cause myocarditis or pericarditis? How should patients with a history of myocarditis or pericarditis be managed? How about patients with a history of MIS-C?

A rare adverse event of myopericarditis has been reported following mRNA vaccination, typically after the second dose and within a week of vaccination. Prevalence is highest in adolescent and young adult males (age 12-29 years old). There are approximately 2000 cases reported to date in the United States, an estimated 5 to 10 cases per 1 million second doses overall and 40 cases per 1 million second doses in males 12 to 29.^8^ Fortunately, almost all affected teens with vaccine-associated myocarditis responded well to medical therapy. Population studies have demonstrated that the risk of COVID-19-related myocarditis is approximately 4 times higher than the risk of vaccine-associated myocarditis in all age groups.^8–10^

Collectively, these data support that the benefit of COVID-19 vaccination outweighs the small risk of myocarditis in all age groups. Patients, particularly adolescent males, should be counseled on the potential and symptoms of myocarditis. For patients who develop myocarditis or pericarditis after a dose of an mRNA COVID-19 vaccine, administration of the subsequent dose of the mRNA vaccine is not recommended.

There are no data related to vaccination during or after nonvaccine-related myocarditis or pericarditis (ie, myopericarditis of other etiologies). A prior history of myocarditis or pericarditis is not an absolute contraindication to vaccination, but it is recommended that vaccination be deferred until cardiac inflammation resolves. Based on expert consensus, a recent episode of multisystem inflammatory syndrome in children (MIS-C) is also not a contraindication to vaccination. Current recommendations are to wait approximately 90 days after MIS-C before administering COVID-19 vaccination because mild heart inflammation can be associated with MIS-C.

https://www.cdc.gov/vaccines/acip/meetings/downloads/slides-2021-11-2-3/04-COVID-Oster-508.pdf

https://www.cdc.gov/vaccines/covid-19/clinical-considerations/covid-19-vaccines-us.html#Contraindications

5. What is the role for testing asymptomatic patients prior to cardiac surgery?

Asymptomatic screening has two potential benefits: (1) detection of early or mild disease in patients who could be at risk for worsening peri-operatively and (2) identification of patients who are infectious to facilitate appropriate transmission-based precautions or case delay to limit healthcare worker exposures.^11,12^ Given the current incubation period of the predominant Omicron variant circulating, screening tests are most beneficial when obtained within 2 to 3 days prior to surgery and less useful more than 5 days prior.

6. What type of test is most appropriate?

PCR tests detect any amount of viral genetic material and are the most sensitive test for detecting SARS-CoV-2.^12^ However, a PCR test may stay positive for weeks to months after an active infection, particularly in younger children or immunocompromised patients. PCRs may continue to be positive long after active infection has resolved, and the patient is no longer contagious. Thus, PCR tests are less useful when a patient has had a recent COVID-19 infection. Experts generally recommend against re-testing with a PCR test within 90 days unless new symptoms (indicating potential re-infection) have developed.

Antigen tests, including most home COVID-19 tests, detect proteins from the SARS-CoV-2 virus, so are more likely to detect living “active” virus.^13^ They are less sensitive than PCR tests, meaning that they have a higher chance of a false negative or missing a current infection. However, they generally work well when combined with a high pre-test probability for detecting active infection—for example, when collected in a symptomatic individual. A positive antigen test is thought to correlate with viral transmissibility. Symptom and time-based strategies to end isolation are favored, but antigen tests may also have utility because patients no longer antigen positive are less likely to be infectious.

Serologic or antibody testing is effective for detecting prior infection (nucleocapsid serology) as well as prior vaccination or infection (spike protein serology). It does not detect active infection, so has little role in preprocedural testing.

https://www.cdc.gov/coronavirus/2019-ncov/hcp/testing-overview.html

7. Are there special considerations for COVID-19 in cardiac transplant patients or other immunocompromised cardiac surgery patients?

Patients who are immunosuppressed may be at higher risk of severe COVID-19. This includes solid organ transplant patients, particularly those who are more severely immunocompromised (eg, those within 3-6 months of transplant or those being treated for rejection), as well as cardiac surgery patients with syndromes or postoperative complications that affect the immune system (eg, DiGeorge Syndrome, chyle leak with associated hypogammaglobulinemia). Vaccine recommendations may differ for these immunocompromised individuals and the most updated CDC guidance should be followed regarding additional doses in the primary series and/or booster doses. Additionally, recent data supports completing COVID-19 vaccination pretransplant, when possible, because immunogenicity of vaccines may not be as robust posttransplant due to immunosuppression.^14^

Even if a patient has received all recommended vaccines, it is still possible that they will be infected with SARS-CoV-2, and this may occur more often in solid organ transplant recipients compared to the general population. In this case, cardiac transplant patients may be candidates for several treatments which target high-risk individuals, including monoclonal antibody therapy (see treatment options below in question 8).^15^

8. What are the considerations for newborns born to mothers with COVID-19?

Perinatal transmission of SARS-CoV-2 is rare, estimated to occur in 2% to 4% of neonates born to an infected mother.^16^ When perinatal transmission occurs, it generally occurs in the postnatal period via infectious respiratory droplets. Transplacental or intrapartum transmission of SARS-CoV-2 is exceedingly rare but has been reported. The most common symptom among infected neonates remains respiratory distress.

Most infants born to SARS-CoV-2 positive mothers do not need to be separated from the infected mother/family. However, it may be reasonable to separate infants with congenital heart disease who are expected to require repair within the first 1 to 2 weeks of life to avoid the risk of them acquiring COVID-19. The choice regarding separation of infant–mother dyads should be an individualized decision made by the local surgical and medical teams, in collaboration with the affected families. If not separated, the American Academy of Pediatrics recommends that infectious mothers mask and perform hand hygiene when providing hands-on care to the newborn. Breastfeeding (masked) or feeding with maternal breastmilk is permitted and encouraged in all scenarios. Breastmilk from infectious individuals does not transmit SARS-CoV-2 and has been shown to have anti-SARS-CoV-2 antibodies.

https://www.aap.org/en/pages/2019-novel-coronavirus-covid-19-infections/clinical-guidance/faqs-management-of-infants-born-to-covid-19-mothers/

9. What are the current treatment options for COVID-19? (updated)

The majority of children infected with SARS-CoV-2 experience a mild illness and recover uneventfully with supportive care only. However, some cardiac surgery patients may be at sufficiently high risk that even mild illness warrants consideration of treatment to slow potential progression. Others may develop illness severe enough to warrant hospitalization, in which case therapy is often indicated. There are several different categories of treatments for children with COVID-19 including (1) passive immunization (ie, antibody therapies, typically monoclonal), (2) antivirals, and (3) immunomodulatory therapies.^17^

### Passive Immunization (Antibody Therapies)

For patients with mild illness able to be managed in the outpatient setting, administration of monoclonal antibodies can be considered in select high-risk patients. There are several monoclonal antibodies that bind to distinct but overlapping regions of the SARS-CoV-2 spike protein, preventing the virus from attaching to human cells. Clinical trials have demonstrated consistent reductions in hospitalization with monoclonal antibody therapy. Because of this mechanism, they are most effective when given very early in the course of illness, when viral replication is highest. Multiple monoclonal antibodies are authorized under FDA Emergency Use Authorization (EUA). However, as of January 2022, only one monoclonal antibody, called sotrovimab, has activity against the Omicron variant. Sotrovimab was studied in a randomized trial including unvaccinated adults with comorbid medical conditions (eg, obesity, diabetes, and hypertension) and was administered to participants within 5 days of symptom onset. Treatment with sotrovimab was associated with an approximately 85% relative risk reduction in hospitalization or death.^18^ While these data are promising, direct extrapolation to children is challenging. Sotrovimab is currently authorized only for patients ≥12 years old and ≥40 kg who are at high risk for progression to severe illness. Sotrovimab supply is extremely limited in the United States, so available drug is often allocated preferentially to older adults at the highest risk.

Convalescent plasma from recovered patients is also available under EUA for children of all ages but there is limited evidence to date of improved outcomes, with the most promising data supporting use very early after infection in severely immunocompromised adults. Very few blood banks are supplying convalescent plasma as of January 2022.

### Antivirals

Nirmatrelvir/ritonavir is an oral protease inhibitor combination antiviral with EUA for outpatient children (≥12 yo and >40 kg) at high risk of progression to severe COVID-19; initiation is recommended within 5 days of COVID-19 symptom onset. This drug had reported efficacy for decreasing severity in pharmaceutical-sponsored adult trials but this data has not yet been published with peer review (https://www.fda.gov/media/155050/download). Notably, the ritonavir component is a strong CYP3A inhibitor and has potentially life-threatening drug interactions with several cardiac medications, including many anti-arrhythmics, amiodarone, tacrolimus, or warfarin. Molnupiravir is a nucleoside analog that inhibits viral replication, and it is authorized under an FDA EUA for outpatients  ≥18 years old at risk of severe disease progression in situations where no alternative treatment (eg, sotrovimab or nirmatrelvir/ritonavir) is available. Molnupiravir is substantially less efficacious in preventing severe disease than other available therapies and has significant toxicities including teratogenicity; it is not available for children due to potential bone and cartilage toxicity.

In our prior FAQ articles, we wrote about remdesivir, an intravenous antiviral medication which is FDA approved for adolescents (≥12 yo) and has now also been granted EUA for younger children (≤12 yo and ≥3.5 kg). Remdesivir seems to shorten the duration of illness among hospitalized patients with hypoxia and may slow progression to respiratory failure. It is the most commonly used inpatient COVID-19 antiviral at this time, including in hospitalized children, though to date there remains limited pediatric evidence.^19^ Additionally, a trial published in December 2021 demonstrated a reduction in hospitalization among high-risk adult outpatients treated with remdesivir which led to some enthusiasm for remdesivir in outpatients.^20^ However, the risk/benefit ratio of outpatient remdesivir in children is in outpatient children and has significant logistical challenges.

### Immunomodulatory Therapies

Dexamethasone continues to be the first-line immunomodulatory therapy for hospitalized children with severe COVID-19 with significant hypoxia and/or invasive or noninvasive mechanical ventilation, including patients on high-flow nasal cannula. This practice is based on data from the RECOVERY trial, which demonstrated a reduction in mortality with dexamethasone therapy in adults requiring supplemental oxygen or respiratory support.^21^

Two other immunomodulatory therapies may be considered for hospitalized children with severe COVID-19: tocilizumab (an IL-6 receptor blocker monoclonal therapy) and barcitinib (a JAK inhibitor). Consultation with pediatric subspecialists comfortable with the use of immunomodulatory medications is suggested. Two randomized trials have demonstrated that tocilizumab may reduce both mortality and severity of disease in critically ill adults, particularly those with significant inflammation.^22,23^ Prior to these studies, multiple trials conducted in hospitalized patients who were less severely ill demonstrated no benefit from tocilizumab, suggesting that efficacy is limited to a subset of critically ill patients. Despite a lack of pediatric evidence, some experts use tocilizumab in critically ill children with COVID-19, particularly those with evidence of inflammation despite dexamethasone. In part this is because tocilizumab is commonly used for several non-COVID-19 pediatric inflammatory conditions, so adverse effects and toxicity are understood. Baricitinib was studied in combination with remdesivir initially; the combination of baricitinib and remdesivir reduced time to clinical recovery in hypoxic adults compared to the use of remdesivir alone.^24^ Subsequently, baricitinib was compared to standard care in hypoxic adults and demonstrated a reduction in mortality in the baricitinib group.^25^ Baricitinib received EUA status for children ≥2 years of age with COVID-19, and may be considered in critically ill children with escalating respiratory support needs.

IDSA Guidelines on the Treatment and Management of Patients with COVID-19 (idsociety.org)

https://www.covid19treatmentguidelines.nih.gov/special-populations/children/

https://www.aap.org/en/pages/2019-novel-coronavirus-covid-19-infections/clinical-guidance/outpatient-covid-19-management-strategies-in-children-and-adolescents/

10. Are the Delta and Omicron variants worse or more severe in pediatric populations than prior SARS-CoV-2 variants?

Although there have been more children hospitalized during the Delta and Omicron surges both globally and in the United States, expert consensus is that disease caused by these variants is not necessarily more severe in children than prior variants. Prevalence of infection of any severity has been higher during these variant surges, so it is not surprising that the absolute number of pediatric hospitalizations is also higher, including children requiring critical care. This increase may be in part due to increasing transmission and contagiousness of Delta and Omicron variants. Further, children are less likely than adults to be vaccinated and therefore remain more vulnerable to infection. In many adult vaccinated populations, omicron has had a milder disease course than prior waves. This is encouraging and likely to also be true in children, though we have less evidence to date; it is another reason to encourage pediatric vaccination.

https://publications.aap.org/

11. What PPE is needed for COVID-19, and is it different for different variants? (updated)

Our knowledge of respiratory disease transmission continues to grow throughout the pandemic, including understanding of appropriate PPE (personal protective equipment). SARS-CoV-2 is transmitted via a combination of larger infectious droplets and smaller infectious aerosols, which exist on a spectrum. Transmission generally occurs within 6 feet of an individual, though aerosolization can occur beyond that distance, particularly during certain procedures (eg, intubation, bag-mask ventilation) often termed “aerosol-generating procedures.” General PPE advice during the first year of the pandemic included surgical masks and eye protection with N95 or respirators are recommended for aerosol-generating procedures. This worked well to prevent transmission in most healthcare settings and the community. During 2021, we have seen variants emerge with higher transmissibility, and, in response, many healthcare systems have expanded the indications for N95/respirator protection. Currently, the CDC recommends the use of either N95s or well-fitted surgical masks while caring for known COVID-positive patients or persons under investigation (PUIs) for COVID-19. Choice of a mask may in part be based on the types of care being performed (eg, intubation), patient or healthcare worker specifics (such as vaccination status), and facility parameters like ventilation. Eye protection should be worn at all times during COVID-19 care. While cloth masks may be more comfortable and are often worn in a nonhospital setting, they are less effective than medical-grade masks and should not be used for clinical care. Guidance continues to evolve, and we recommend following local infection control guidelines which may be based, in part, upon local COVID-19 prevalence.^17^

In terms of patient and community protection, the best way to prevent respiratory disease transmission is to employ a “layering” approach. Layers of protection may include vaccination, ventilation, distancing, and masking in certain settings.

https://www.cdc.gov/coronavirus/2019-ncov/hcp/infection-control-recommendations.html

## Summary

The dreadful SARS-CoV-2 virus continues to create exceptional challenges at all levels—public sector, hospitals, and the entire healthcare industry. We are now more adept at evaluating and prioritizing (COVID-19 and non-COVID-19) patient problems and have established standardized management algorithms. Collaboration between the surgical and medical specialties has been exemplary. Leadership in our specialty is about bringing order to chaos and fighting uncertainty…and that is what our specialty is doing collectively. As the virus and pandemic evolve, the medical profession evolves. We are living in unprecedented times which have presented often difficult dilemmas. Who gets surgery and who should be delayed? Can family members visit their ailing child or sibling? When and how best to test? How long to quarantine? Some of these questions have been answered and some will continue to change. Regardless, we will adapt and make decisions based on the accumulation of scientific evidence to provide the best possible care to our patients during this inimitable time.
